# A Case of Nutritional Optic Neuropathy Caused by Vitamin B12 and Folate Deficiency Presenting With Bitemporal Hemianopia-Like Visual Field Defects

**DOI:** 10.7759/cureus.109561

**Published:** 2026-05-24

**Authors:** Naoto Ueda, Yuta Kitamura, Takayuki Baba

**Affiliations:** 1 Ophthalmology, Chiba University, Chiba, JPN

**Keywords:** bitemporal hemianopia‐like visual field defect, folate deficiency, nutritional optic neuropathy, smoking, unbalanced diet, vitamin b12 deficiency

## Abstract

Nutritional optic neuropathy is a rare condition caused by vitamin B12 or folate deficiency. It typically presents with subacute bilateral vision loss and central visual field defects, whereas a presentation resembling bitemporal hemianopia is uncommon. A 70-year-old man living alone presented with gradually worsening bilateral vision loss over four months. His best-corrected visual acuity (BCVA) had decreased to 20/400 in the right eye (OD) and 20/200 in the left eye (OS). Optical coherence tomography showed widespread thinning of the ganglion cell complex, with preservation of the peripapillary retinal nerve fiber layer. Humphrey 30-2 perimetry revealed bitemporal hemianopia-like defects, raising suspicion of a chiasmal lesion; however, contrast-enhanced MRI was normal. Serum vitamin B12 and folate levels were low (66 pg/mL and 3.4 ng/mL), leading to a diagnosis of nutritional optic neuropathy. The patient started oral methylcobalamin (1.5 mg/day) and folic acid (10 mg/day), along with smoking cessation counseling. After three weeks of treatment, vitamin levels normalized, and approximately 15 weeks later, BCVA improved to 20/30 in OD and 20/40 in OS, with significant recovery of visual fields. Nutritional optic neuropathy may rarely cause bitemporal hemianopia-like visual field defects. In patients with subacute bilateral vision loss, assessing diet and smoking history, along with measuring vitamin B12 and folate levels, is important.

## Introduction

Nutritional optic neuropathy results from insufficient intake or impaired absorption of vitamin B12, folate, or other essential nutrients. Vitamin B12 and folate are essential cofactors in one-carbon metabolism and mitochondrial energy production; their deficiency impairs myelin synthesis and mitochondrial function, leading to optic nerve damage [1,2]. Clinically, it manifests with painless, bilateral, subacute vision loss, color vision deficits, and central or cecocentral scotomas [1]. Optical coherence tomography (OCT), a non-invasive retinal imaging technique, typically shows early thinning of the ganglion cell complex (GCC), the innermost retinal layers containing the cell bodies and proximal axons of retinal ganglion cells, while thinning of the peripapillary retinal nerve fiber layer (RNFL), which reflects the more distal axonal segments, may occur later in the disease course [3].

We report a case that initially exhibited bitemporal hemianopia-like visual field defects, raising concerns for a chiasmal lesion, but was ultimately diagnosed with nutritional optic neuropathy related to vitamin B12 and folate deficiency in the context of an unbalanced diet and long-term smoking. What makes this case unusual is that such bitemporal hemianopia-like defects, a pattern typically associated with compression of the optic chiasm by lesions such as pituitary adenomas, are a rare and atypical manifestation of nutritional optic neuropathy, which more commonly presents with central or cecocentral scotomas. This case highlights the importance of including nutritional optic neuropathy in the differential diagnosis of subacute bilateral visual loss, even when the visual field pattern atypically mimics a chiasmal lesion.

## Case presentation

A 70-year-old man living alone, with a history of diabetes mellitus but no diabetic retinopathy, presented with a four-month history of gradually worsening bilateral blurred vision. He had previously maintained normal visual acuity (best-corrected visual acuity (BCVA) 20/20 in both eyes (OU)) and had no history of exposure to medications known to cause optic neuropathy.

Initial evaluation (onset to two months)

**Figure 1:**
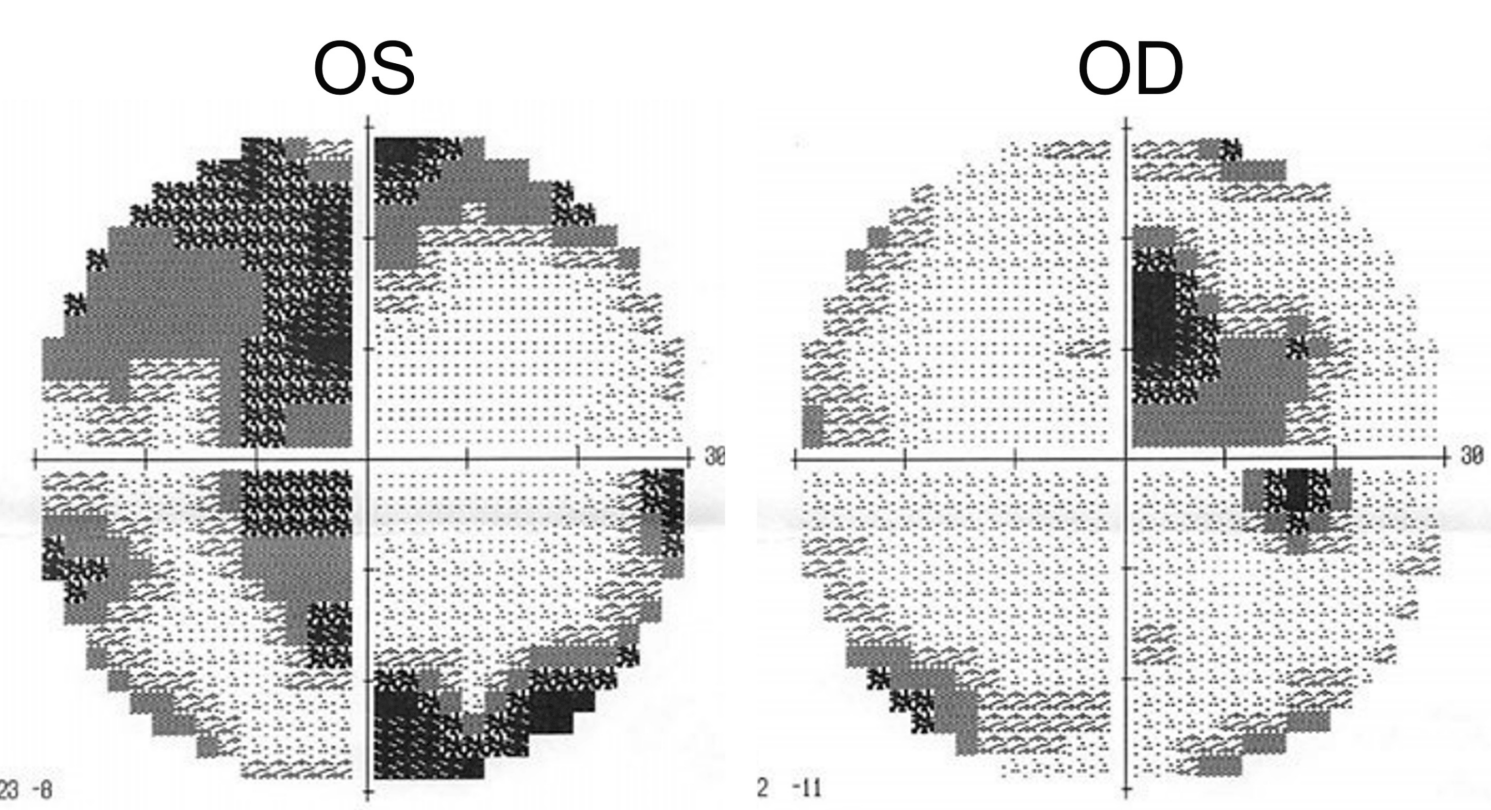
Humphrey visual field test (30-2) performed two months after the onset of subjective visual acuity loss Bitemporal hemianopia-like visual field defects are observed. OD: fixation losses 1/1, false-positive errors 8%, false-negative errors 16%, VFI 76%, MD −7.84 dB, and PSD 6.93 dB. OS: fixation losses 0/0, false-positive errors 5%, false-negative errors 26%, VFI 65%, MD −11.26 dB, and PSD 9.41 dB. OD: right eye, VFI: visual field index, MD: mean deviation, PSD: pattern standard deviation, OS: left eye

At the onset of symptoms, BCVA was 20/32 OU, declining to 20/200 OU over the subsequent two months. OCT demonstrated diffuse macular GCC thinning with preserved RNFL thickness bilaterally. Humphrey 30-2 automated perimetry revealed bitemporal hemianopia-like visual field defects (Figure 1), raising suspicion for a chiasmal lesion. However, contrast-enhanced MRI showed no abnormalities around the optic chiasm or pituitary gland, making a structural chiasmal etiology unlikely.

Referral evaluation (approximately four months after onset)

The patient was referred to our ophthalmology department for further evaluation. On presentation, BCVA had further declined to 20/400 in the right eye (OD) and 20/200 in the left eye (OS). There was no ocular pain, and refraction revealed mild hyperopic astigmatism bilaterally. No neurological symptoms, such as limb numbness or gait disturbance, were present.

**Figure 2:**
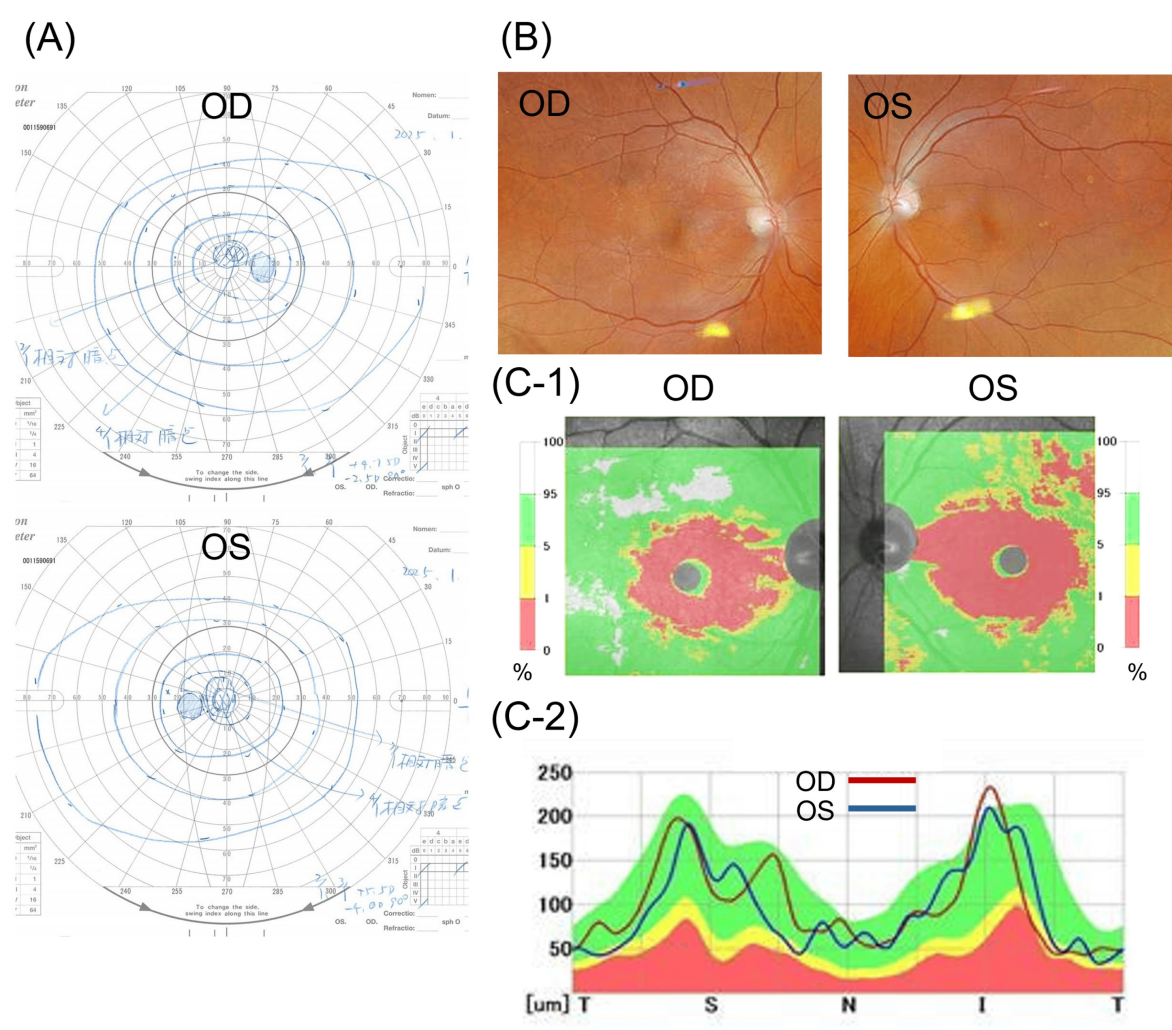
Findings obtained approximately four months after the onset of subjective visual acuity loss (A) Goldmann perimetry demonstrated a central relative scotoma. (B) Fundoscopy revealed mild bilateral optic disc pallor. (C) OCT findings at the initial visit: (C-1) GCC deviation map (measured from the ILM to the IPL/INL boundary). The color bar represents GCC thickness as a percentage relative to the age-matched normative database: red, below the 1st percentile; yellow, 1st-5th percentile; and green, within normal limits (>5th percentile). Diffuse GCC thinning is predominantly observed in the central macular region bilaterally. (C-2) The RNFL thickness profile graph shows the peripapillary scan position by clock-hour sector (T, S, N, and I) on the x-axis and RNFL thickness (μm) on the y-axis. The green-shaded area denotes the normal range of the age-matched normative database, whereas the yellow and red areas indicate the 1st-5th percentile range and below the 1st percentile, respectively. RNFL thickness is relatively preserved bilaterally. OD: right eye, OS: left eye, OCT: optical coherence tomography, GCC: ganglion cell complex, ILM: inner limiting membrane, IPL/INL: inner plexiform layer/inner nuclear layer, RNFL: retinal nerve fiber layer, T: temporal, S: superior, N: nasal, I: inferior

Critical flicker frequency (CFF), a measure of visual pathway function, was markedly reduced to <1.0 Hz OD and 6.8 Hz OS (normal >30 Hz). Intraocular pressure was within normal limits bilaterally, and the anterior segment was unremarkable. Goldmann visual field testing at this visit demonstrated central relative scotomas (Figure 2A), a pattern more typical of nutritional optic neuropathy, differing from the bitemporal pattern observed on earlier automated perimetry. Fundoscopy revealed mild bilateral optic disc pallor (Figure 2B). OCT confirmed diffuse macular GCC thinning with preserved RNFL thickness in OU (Figure 2C).

Dietary and social history

Detailed history-taking revealed that the patient lived alone and subsisted primarily on simple self-prepared meals. Following an episode of urticaria four years earlier, he had avoided blue-backed fish, shellfish, and raw eggs, all of which are important dietary sources of vitamin B12. He had smoked approximately 20 cigarettes per day for 50 years. Smoking is known to impair folate metabolism and to increase oxidative stress. He had abstained from alcohol for the past 20 years.

Laboratory findings (two weeks after referral)

Blood tests revealed markedly reduced serum vitamin B12 at 66 pg/mL and folate at 3.4 ng/mL, both below the normal reference ranges (vitamin B12: 180-914 pg/mL; folate: 3.6-12.9 ng/mL). Hemoglobin (14.9 g/dL) and mean corpuscular volume (MCV, 93.6 fL) were within normal limits, indicating the absence of overt megaloblastic anemia. The patient had no history of gastrointestinal disease, prior gastrectomy, or chronic use of proton pump inhibitors or metformin, suggesting that the deficiency was attributable to inadequate dietary intake rather than malabsorption. Although endoscopy was recommended to exclude gastric pathology that could affect intrinsic factor production, the patient declined.

Based on the combination of bilateral subacute vision loss, early GCC thinning with preserved RNFL on OCT, and confirmed vitamin B12 and folate deficiency in the context of a restricted diet and long-term smoking, a diagnosis of nutritional optic neuropathy was established. The clinical course is summarized in Table 1.

**Table 1:** Timeline of clinical findings and treatment course BCVA: best-corrected visual acuity, OU: both eyes, GCC: ganglion cell complex, OCT: optical coherence tomography, MRI: magnetic resonance imaging, OD: right eye, OS: left eye, CFF: critical flicker frequency

Time from onset	Clinical course
0 month	Blurred vision; BCVA 20/32 OU
2 months	BCVA 20/200 OU; GCC thinning on OCT; bitemporal defects on Humphrey 30-2; MRI normal
4 months	BCVA 20/400 OD, 20/200 OS; central scotomas; CFF <1.0 Hz OD, 6.8 Hz OS; smoking cessation counseling
+2 weeks	Low vitamin B12 and folate; methylcobalamin and folic acid initiated
+3 weeks	Vitamin levels normalized
+12 weeks	BCVA 20/30 OD, 20/40 OS; visual field markedly improved; GCC thinning persisted

Differential diagnosis

The initial bitemporal hemianopia-like defects strongly suggested a chiasmal disorder, such as a pituitary adenoma or craniopharyngioma. However, MRI showed no abnormalities around the optic chiasm, making this diagnosis unlikely. Retinal conditions associated with central visual loss, including age-related macular degeneration, diabetic macular edema, and central serous chorioretinopathy, were considered. Still, the preserved outer retinal layers on OCT and the absence of macular abnormalities ruled them out.

Hereditary optic neuropathies, such as Leber hereditary optic neuropathy, were also considered; however, the patient's older age at onset, lack of family history, and improvement in vision with treatment argued against this diagnosis. Ischemic optic neuropathy and optic neuritis were unlikely due to the bilateral, subacute, painless course, which differs from the typical presentation.

Bilateral symmetric vision loss, early GCC thinning with preserved RNFL, significant vitamin deficiency, and clinical improvement after supplementation align with nutritional optic neuropathy.

Therapeutic intervention

Oral methylcobalamin (1.5 mg/day) and folic acid (10 mg/day) were initiated after confirming deficiency. In the absence of gastrointestinal disease or risk factors for malabsorption, oral therapy was selected over intramuscular injections. Smoking cessation was recommended.

Follow-up and outcomes

**Figure 3:**
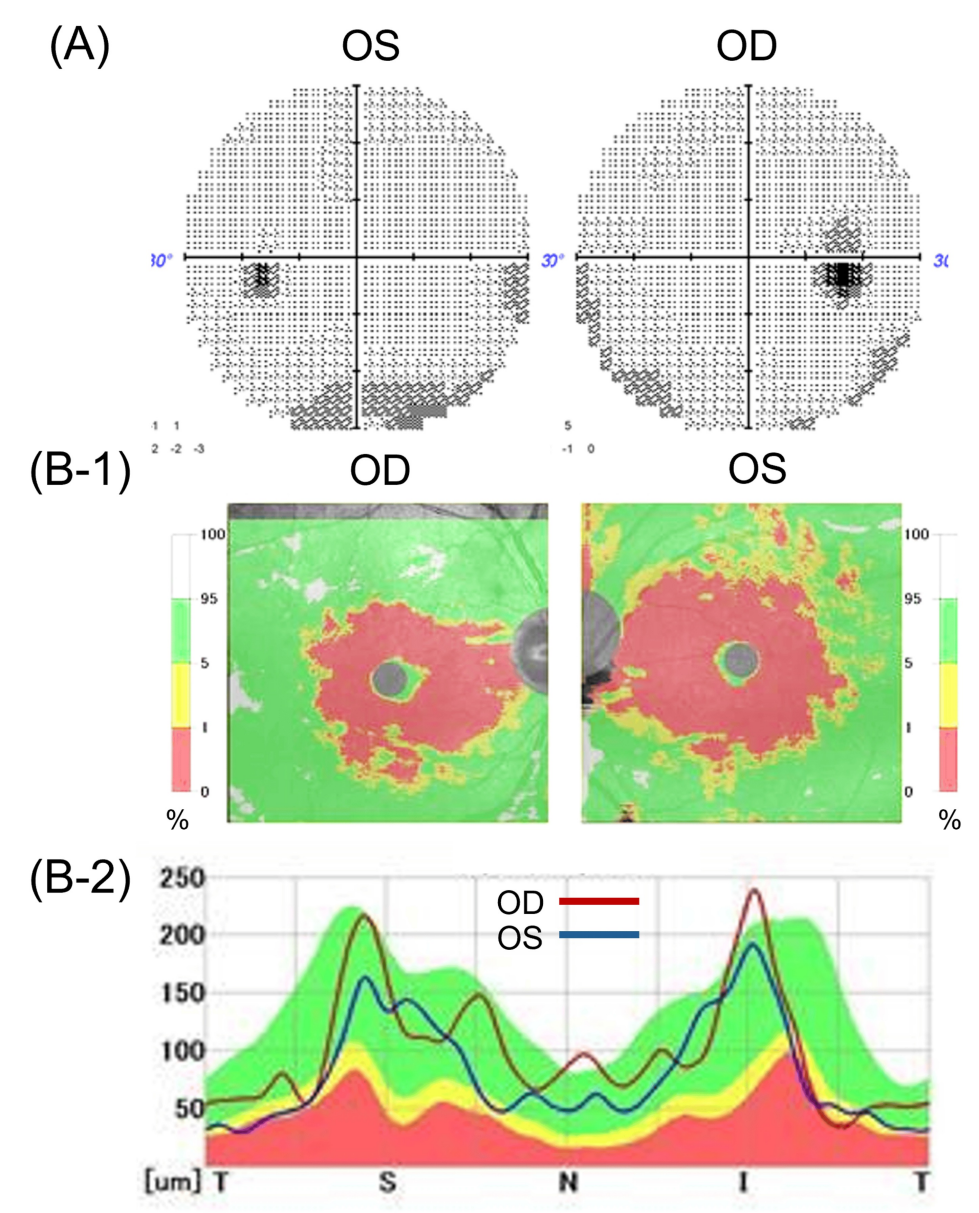
Post-treatment findings (A) Humphrey visual field test (30-2) performed 12 weeks after treatment showed significant improvement in central visual field defects. OD: fixation losses 9/13, false-positive errors 0%, false-negative errors 11%, VFI 93%, MD −3.32 dB, PSD 3.62 dB. OS: fixation losses 8/13, false-positive errors 7%, false-negative errors 18%, VFI 93%, MD −3.53 dB, PSD 2.61 dB. (B) OCT findings at 13 weeks post-treatment: (B-1) GCC deviation map (ILM to IPL/INL). The color bar represents GCC thickness as a percentage relative to the age-matched normative database using the same color coding as in Figure 2C-1: red indicates values below the 1st percentile, yellow indicates values between the 1st and 5th percentiles, and green indicates values within normal limits. Diffuse GCC thinning persists in the central macular region bilaterally despite significant recovery of visual function. (B-2) Peripapillary RNFL thickness profile graph. The x-axis represents the peripapillary scan position by clock-hour sector (T, S, N, and I), and the y-axis represents RNFL thickness (μm). The red and blue lines indicate the OD and OS, respectively. The green-shaded area denotes the normal range of the age-matched normative database, whereas the yellow and red areas indicate the 1st-5th percentile range and below the 1st percentile, respectively. RNFL thickness remains relatively preserved bilaterally, consistent with the initial presentation findings. OD: right eye, OS: left eye, VFI: visual field index, MD: mean deviation, PSD: pattern standard deviation, OCT: optical coherence tomography, GCC: ganglion cell complex, ILM: inner limiting membrane, IPL/INL: inner plexiform layer/inner nuclear layer, RNFL: retinal nerve fiber layer, T: temporal, S: superior, N: nasal, I: inferior

After three weeks of treatment, vitamin B12 and folate levels normalized. Approximately 15 weeks later, BCVA improved to 20/30 OD and 20/40 OS, with significant improvement in central visual field sensitivity (Figure 3A). GCC thinning continued, and RNFL thickness remained stable (Figure 3B). No adverse events were observed.

## Discussion

Nutritional optic neuropathy results from mitochondrial metabolic impairment due to vitamin B12 and folate deficiency, typically affecting the papillomacular bundle [1]. Previous studies have indicated that thinning of the GCC on OCT may precede changes in the RNFL [2], and our findings support this conclusion, as diffuse GCC thinning was observed while the RNFL remained relatively intact at presentation.

In nutritional optic neuropathy, central or cecocentral scotomas are commonly reported [1], and bitemporal hemianopia-like defects are considered very rare. In our case, the later Goldmann perimetry revealed a typical central relative scotoma. Regarding the mechanism underlying the initial bitemporal pattern, several hypotheses can be considered: (1) reduced reliability of the initial automated perimetry, including false negatives or lack of familiarity with the test; (2) a possible mechanism of early metabolic dysfunction mainly affecting fibers contributing to the nasal retina, as the papillomacular bundle is mainly composed of small-caliber retinal ganglion cell axons that are metabolically vulnerable because of their unfavorable surface-to-volume ratio and limited mitochondrial reserve [3], and histopathological studies in mitochondrial optic neuropathies, such as Leber hereditary optic neuropathy, have shown that small-caliber axons are preferentially affected [4], suggesting that fibers entering the temporal optic disc along the disc-fovea axis may be selectively involved in the early stage; and (3) early preferential involvement of the chiasm itself, as Sharma and Sharma have noted that the primary lesion in toxic/nutritional optic neuropathy has not been definitively localized to the optic nerve and may originate at the level of the retina, chiasm, or optic tracts [5].

Regarding background factors, the patient lived alone and had a long-term limited intake of certain foods, such as blue-backed fish, shellfish, and raw eggs, along with a history of smoking. These factors may have combined to cause vitamin B12 and folate deficiency [6,7]. Since dietary habits are often difficult to fully assess during routine history taking, detailed questions about nutrition and lifestyle are essential when nutritional optic neuropathy is suspected.

In this case, visual recovery occurred within a relatively short period after correction of the vitamin deficiency. It has been suggested that a longer duration of deficiency may cause irreversible optic nerve damage [1], and the positive outcome in this case might be due to supplementation starting within several months of symptom onset. Conversely, the continued presence of GCC thinning on OCT after visual function improved indicates that functional and structural recovery do not necessarily happen simultaneously.

Limitations

First, this case lacked evaluation for an endoscopic assessment or intrinsic factor because the patient refused, leaving malabsorption not fully ruled out. Second, the follow-up period was relatively short, and long-term structural recovery remains unknown. Third, the cause of early bitemporal defects cannot be definitively determined due to limited visual field data.

## Conclusions

We report a case of nutritional optic neuropathy due to vitamin B12 and folate deficiency that initially presented with bitemporal hemianopia-like visual field defects, an atypical and rarely described manifestation of this condition. This presentation led to initial concern for a chiasmal lesion; however, normal MRI findings and confirmed vitamin deficiency ultimately established the correct diagnosis. Following oral vitamin supplementation, significant recovery of visual function was achieved.

The key clinical teaching point of this case is that nutritional optic neuropathy should be included in the differential diagnosis of subacute bilateral visual loss, even when the visual field pattern atypically mimics a chiasmal lesion. In such patients, a thorough assessment of dietary habits and smoking history, along with measurement of serum vitamin B12 and folate levels, is essential and may prevent diagnostic delay. Early diagnosis and prompt supplementation are critical, as prolonged deficiency risks irreversible optic nerve damage.
